# Diagnostic evaluation of renal masses in chronic kidney disease: a clinical and radiologic challenge

**DOI:** 10.1007/s00261-026-05491-4

**Published:** 2026-04-10

**Authors:** Laura Eusebi, Federica Masino, Manuela Montatore, Valentina Testini, Giuseppe Guglielmi

**Affiliations:** 1 Radiology Unit, “Carlo Urbani” Hospital, Jesi, AN Italy; 2 Radiology Unit, “Vittorio Emanuele II” Hospital, Bisceglie, BT Italy; 3 Department of Clinical and Experimental Medicine, Foggia University School of Medicine, Foggia, FG Italy; 4 Radiology Unit, “Dimiccoli” Hospital, Barletta, BT Italy; 5 Radiology Unit, “IRCCS Casa Sollievo della Sofferenza”, San Giovanni Rotondo, FG, Italy

**Keywords:** Chronic kidney disease, Renal mass, Diagnostic imaging, Contrast-enhanced ultrasound, Contrast agents

## Abstract

Chronic kidney disease (CKD) increases the risk of renal cell carcinoma (RCC), particularly with long-term dialysis exposure and acquired cystic kidney disease (ACKD). At the same time, CKD-related architectural distortion and hypoperfusion reduce lesion conspicuity on ultrasound and weaken enhancement-based interpretation on CT and MRI. In advanced CKD, restrictions on iodinated and gadolinium-based contrast agents further increase the proportion of renal masses that remain indeterminate after conventional imaging. Contrast-enhanced ultrasound (CEUS) addresses the key unmet need in this setting: a safe, direct assessment of microvascular perfusion. Microbubble agents are purely intravascular and non-nephrotoxic, enabling use across all CKD stages and in dialysis patients. By showing real-time enhancement of septa, wall thickening and mural nodules, CEUS distinguishes vascular tumour components from avascular hemorrhagic or proteinaceous material that can mimic enhancement on CT/DECT or signal abnormalities on non-contrast MRI. This capability supports a CEUS-adapted Bosniak approach: lesion categorization is driven by enhancement of septa, wall and nodules rather than by attenuation or T1 hyperintensity of cyst contents, helping avoid misclassification in ACKD. We propose a structured workflow that integrates the triad of CKD stage/eGFR, dialysis status and ACKD with pre- and post-CEUS Bosniak assessment to guide follow-up versus biopsy or nephron-sparing treatment. This article is a narrative review of the literature, complemented by an illustrative clinical case which demonstrates how CEUS can resolve ambiguity when CT/DECT and non-contrast MRI remain inconclusive.

## Introduction

### Chronic kidney disease and the risk of renal tumours

Chronic kidney disease (CKD) is a major global health pathology whose prevalence continues to rise, driven largely by ageing populations and increasing rates of diabetes and hypertension [1]. According to KDIGO 2024, CKD is defined by persistent structural or functional renal abnormalities lasting at least 3 months and is now recognized not only as a systemic condition with cardiovascular implications, but also as a state of increased oncological vulnerability [1, 2]. Indeed, multiple epidemiological studies consistently demonstrate a substantially higher incidence of renal cell carcinoma (RCC) in individuals with CKD, with risk escalating to more than ten-fold among patients undergoing long-term dialysis [3–5].

Acquired cystic kidney disease (ACKD), a condition that develops in the majority of dialysis-dependent patients, represents the strongest substrate for tumor development, with RCC frequently arising within complex cysts and often displaying atypical histological or imaging characteristics compared with RCC in the general population [6–8].

Despite the increased prevalence of renal tumors in CKD, their detection and characterization remain challenging [9, 10]. Altered renal architecture, including cortical thinning, global scarring and loss of corticomedullary differentiation, diminishes lesion conspicuity on ultrasound (US) and can mimic neoplastic processes, particularly in ACKD where multiple cysts with septa, debris or mural irregularities are common [7, 11, 12]. In parallel, chronic reductions in renal perfusion attenuate contrast uptake on Computed Tomography (CT) and Magnetic Resonance Imaging (MRI), complicating application of conventional enhancement criteria and reducing diagnostic confidence [9, 12]. As a result, imaging findings in CKD are disproportionately classified as indeterminate, often prompting additional examinations or biopsy [13].

Choosing the optimal imaging modality is further complicated by safety considerations related to iodinated and gadolinium-based contrast agents [9]. Although recent evidence challenges historical concerns surrounding contrast-associated acute kidney injury, in many centers iodinated contrast use in advanced CKD remains selective rather than routine, particularly in hemodynamically unstable patients, and is typically guided by an individualized risk–benefit assessment and appropriate preventive measures in line with contemporary ACR and ESUR guidance [14]. Similarly, despite the markedly reduced risk of nephrogenic systemic fibrosis with macrocyclic gadolinium agents, gadolinium administration is often limited at low estimated glomerular filtration rate and reserved for scenarios in which the expected diagnostic benefit (e.g., oncological urgency, need for definitive characterization or staging) outweighs residual risk, ideally within a patient- and context-specific decision [9, 15]. These constraints frequently necessitate modified or non-contrast imaging protocols, undermining CT and MRI performance at precisely the moment when accurate characterization is most critical [9].

Early and reliable differentiation of benign from malignant renal lesions is essential for optimizing treatment in CKD, where the balance between oncological control and preservation of residual renal function is particularly delicate [4, 16]. Nephron-sparing surgery, ablative techniques or active surveillance may be preferable to radical nephrectomy; however, these decisions rely on imaging that is both safe and diagnostically robust [17–19]. In this context, newer modalities capable of evaluating microvascularity without nephrotoxic contrast agents, most notably contrast-enhanced ultrasound (CEUS), have gained substantial attention and are increasingly incorporated into recent EFSUMB and ESUR recommendations [20–22].

This manuscript is conceived as a narrative review of the literature, complemented by an illustrative clinical case. Literature was selected through targeted searches of PubMed and recent international guidelines, focusing on imaging of renal masses in chronic kidney disease and contrast-enhanced ultrasound applications. No formal systematic review methodology was performed.

## Diagnostic imaging modalities in chronic kidney disease

The diagnostic evaluation of renal masses in CKD is challenging, and imaging modalities are further limited by restrictions on contrast administration. Building on the pathophysiological framework outlined above, this section details the modality-specific strengths and limitations of US, CT, MRI and renal mass biopsy in CKD [20].

Across all stages of CKD, particularly in advanced disease and dialysis-dependent patients, the kidneys undergo a spectrum of morphological changes including cortical thinning, global scarring, cystic remodeling, interstitial fibrosis and microvascular rarefaction [9, 23]. These abnormalities reduce the intrinsic contrast between cortex and medulla, obscure anatomical landmarks and produce heterogeneous echogenicity on US and altered signal characteristics on CT and MRI. As confirmed in recent imaging guidelines, these changes markedly reduce lesion conspicuity and complicate the assessment of small solid nodules or subtle mural components within cystic lesions [11].

Perfusional impairment is equally consequential. Diminished blood flow and delayed enhancement affect the diagnostic reliability of contrast-based modalities. Enhancement thresholds traditionally used to distinguish benign from malignant lesions may be unreliable in CKD, particularly for hypovascular tumors such as papillary RCC, which are more prevalent in this population. These pathophysiological changes form the substrate for the widespread diagnostic uncertainty encountered when using CT or MRI in CKD without contrast, reduced contrast dose or in settings of compromised renal perfusion [24].

### Ultrasound

**Fig. 1:**
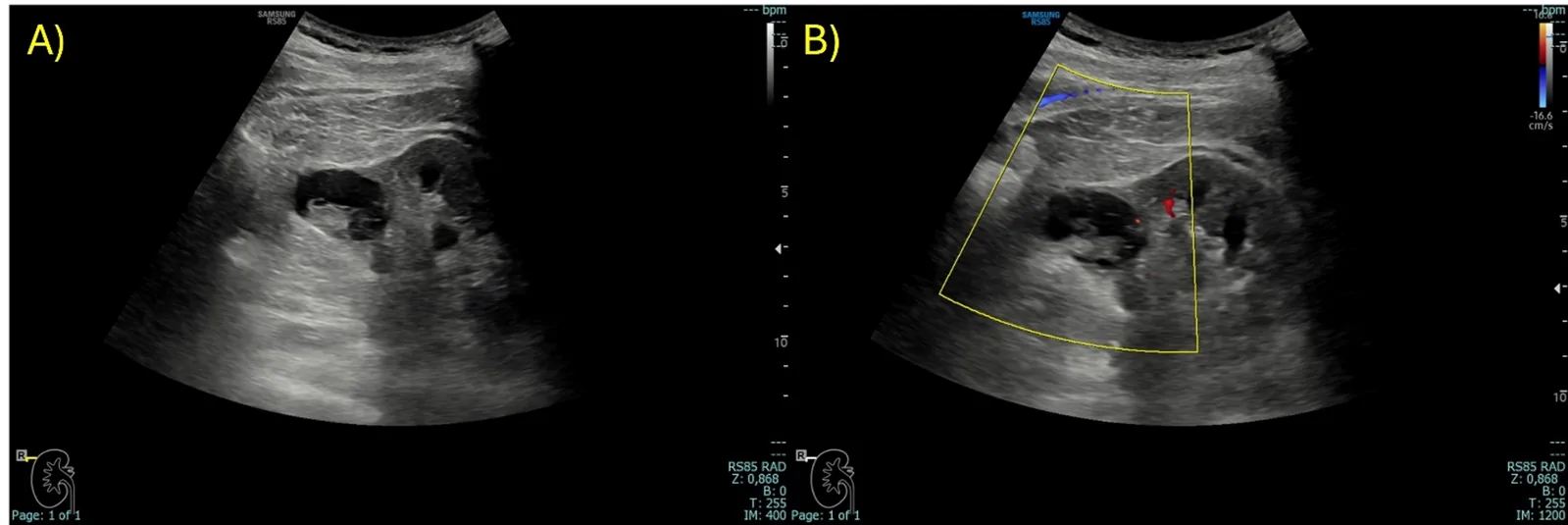
Grey-scale ultrasound images showed an exophytic, predominantly cystic renal lesion located at the right lower renal pole, with internal septa and a mural solid component (**A**). Colour Doppler ultrasound showed no detectable intralesional vascular signal (**B**), in the setting of cortical thinning and heterogeneous renal parenchymal echotexture consistent with chronic kidney disease

Conventional US remains the recommended first-line modality for renal assessment in CKD (ACR Appropriateness Criteria 2023), due to its wide availability, absence of ionizing radiation and independence from renal function. It is particularly useful for confirming CKD-associated morphological alterations, identifying simple cysts and detecting gross masses [11, 13] (Fig. 1).

However, US is limited by the altered echotexture typical of CKD, especially in ACKD where multiple complex cysts are present. These changes reduce the contrast between lesions and background parenchyma, making mural nodules or small solid lesions difficult to detect. Doppler evaluation has low sensitivity in these low-perfusion kidneys, further restricting the modality’s usefulness in characterizing vascularity [25]. Consequently, while US plays a central role in initial assessment and surveillance of ACKD, its ability to reliably characterize indeterminate lesions is limited, necessitating adjunctive imaging.

**Table:** 

**What US can answer in CKD.** Baseline renal morphology, simple versus complex cysts, gross solid masses and surveillance of acquired cystic kidney disease	
**Common US pitfalls in CKD.** Heterogeneous echotexture and cortical thinning reduce lesion conspicuity; complex ACKD cysts with septa/debris may mimic mural nodules; Doppler often underestimates vascularity in hypoperfused kidneys	
**When to escalate to CEUS/biopsy.** Escalate to CEUS for any indeterminate complex cyst or suspected mural/solid component without convincing Doppler flow; reserve biopsy for lesions that remain indeterminate after CEUS or when histology will change management	

### Computed tomography and dual-energy CT

CT, particularly contrast-enhanced CT (CECT), is the gold standard for renal mass assessment in the general population. In CKD, however, its performance is often limited by both contrast policy and altered renal perfusion. In many centers, iodinated contrast use in advanced CKD remains selective rather than routine, especially in patients with additional risk factors such as hemodynamic instability, volume depletion or intercurrent acute kidney injury. As a result, tailored protocols (non-contrast examinations, reduced dose, delayed timing or limited phases) are frequently adopted following a patient- and context-specific risk–benefit assessment, which can compromise diagnostic yield [12, 14].

Second, reduced cortical perfusion and diffuse scarring in CKD kidneys attenuate enhancement, making differentiation between subtle solid components and non-enhancing debris challenging. Cystic lesions in ACKD often contain hemorrhagic or proteinaceous material, leading to hyperdense or complex appearances on non-contrast CT that may artificially elevate Bosniak classification. Conversely, hypovascular RCC subtypes, particularly papillary RCC, may exhibit minimal enhancement even under optimal conditions, increasing the likelihood of false-negative or equivocal examinations [26, 27].

**Table:** 

**What CT can answer in CKD.** Lesion density, calcifications and macroscopic fat; when contrast is feasible, enhancement-based Bosniak assessment and staging (including venous involvement)	
**Common CT pitfalls in CKD.** Attenuated/delayed enhancement from hypoperfusion; hyperdense hemorrhagic/proteinaceous cyst content mimicking enhancement; minimal enhancement in hypovascular subtypes (e.g., papillary RCC) leading to equivocal studies	
**When to escalate to CEUS/biopsy.** Escalate to CEUS when iodinated contrast is contraindicated or enhancement remains equivocal on CT/DECT; consider biopsy if CEUS cannot resolve indeterminacy or if histology is required to guide nephron-sparing treatment versus surveillance	

**Fig. 2:**
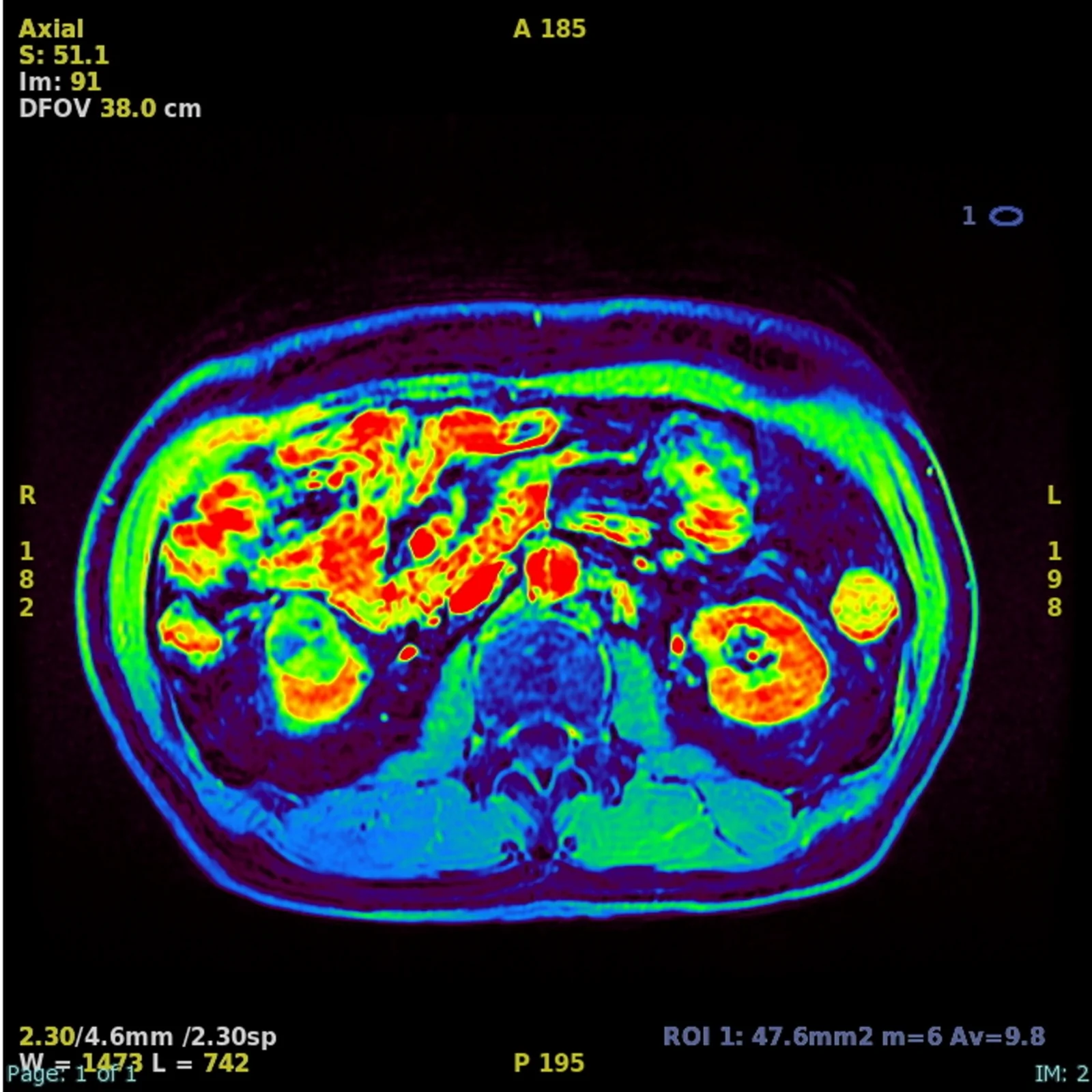
Dual-energy CT iodine map showed a heterogeneous mass at the right lower renal pole, characterised by hyperattenuating cystic content and faint iodine uptake within the mural component

Dual-energy CT (DECT) enhances CT performance by quantifying iodine uptake and improving visualization of subtle enhancement through iodine maps and virtual non-contrast images. DECT has shown particular value for hypovascular tumors and complex cysts. However, it remains dependent on iodinated contrast, and the reduced renal perfusion of CKD still limits the visibility of enhancement. As a result, although DECT may increase confidence compared with conventional CT, it seldom fully resolves ambiguity in advanced CKD [24, 28–30] (Fig. 2).

### Magnetic resonance imaging

MRI offers superior soft tissue contrast and multiparametric assessment without ionising radiation, making it a valuable tool in selected CKD patients. Non-contrast T1- and T2-weighted sequences, diffusion-weighted imaging (DWI) and in selected cases chemical-shift imaging can provide useful insights into lesion architecture. However, MRI is challenged by several CKD-related features: diffuse T1 hypointensity, heterogeneous T2 hyperintensity and loss of corticomedullary differentiation diminish lesion-background contrast. Proteinaceous or hemorrhagic cysts, extremely common in ACKD, may mimic solid enhancing components on non-contrast sequences [9, 15].

DWI is frequently used to support the characterization of indeterminate renal lesions, but its interpretation in CKD is challenging: diffuse interstitial fibrosis and chronic inflammatory change can produce the appearance of diffusion restriction with low apparent diffusion coefficient (ADC) values, potentially mimicking malignancy. This phenomenon reduces the specificity of DWI in CKD kidneys and limits its reliability when contrast-enhanced sequences cannot be performed.

Practical note: in CKD, interpret DWI/ADC as supportive only; avoid upgrading malignancy risk without an enhancement correlate and/or convincing morphology.

**Fig. 3:**
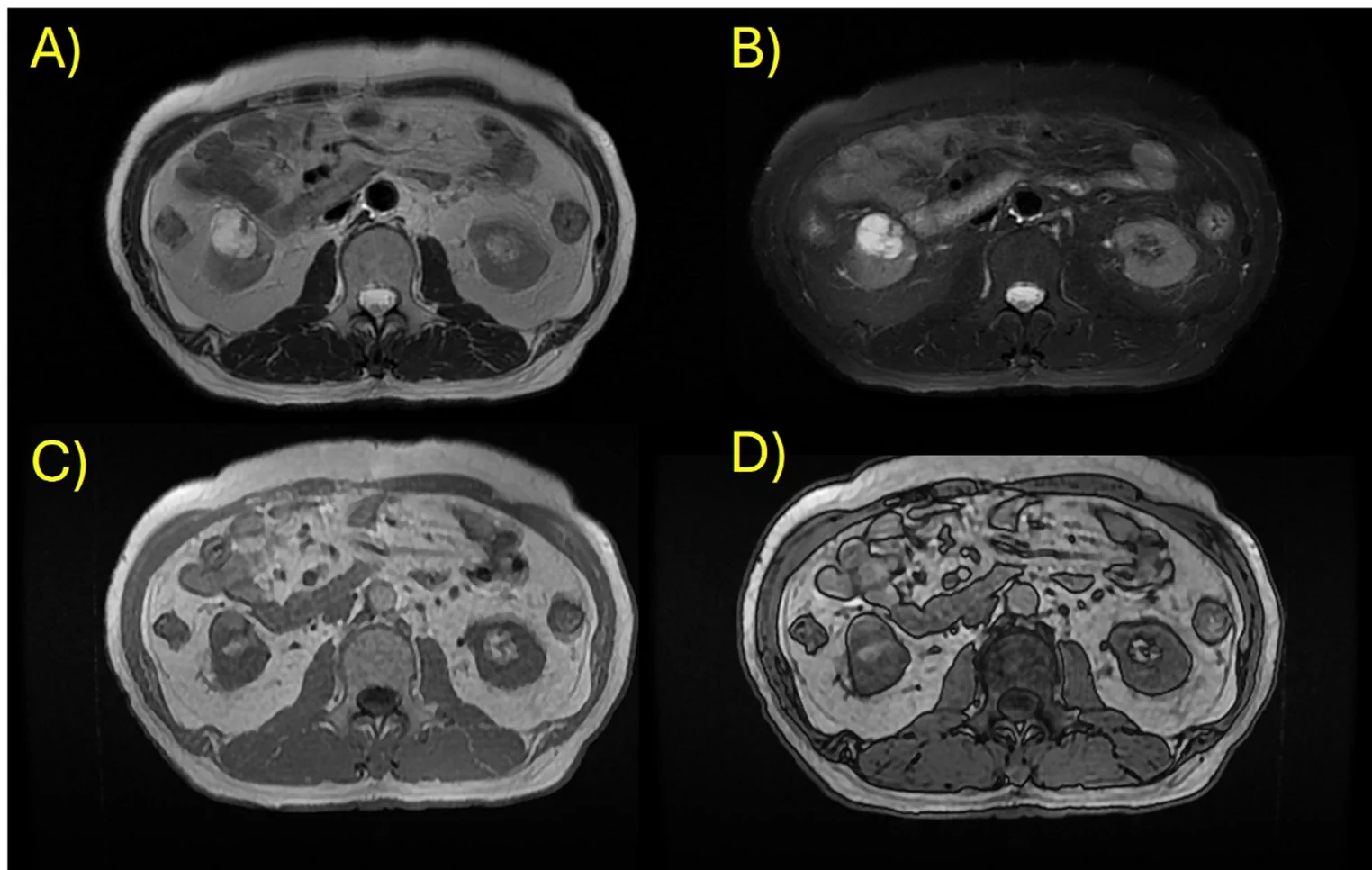
MRI images: axial T2-weighted sequences without (**A**) and with fat suppression (**B**) showed a complex cystic lesion of the right renal lower pole. In-phase and out-of-phase T1-weighted sequences (**C** and **D**) showed no signal drop, indicating absence of macroscopic fat

**Fig. 4:**
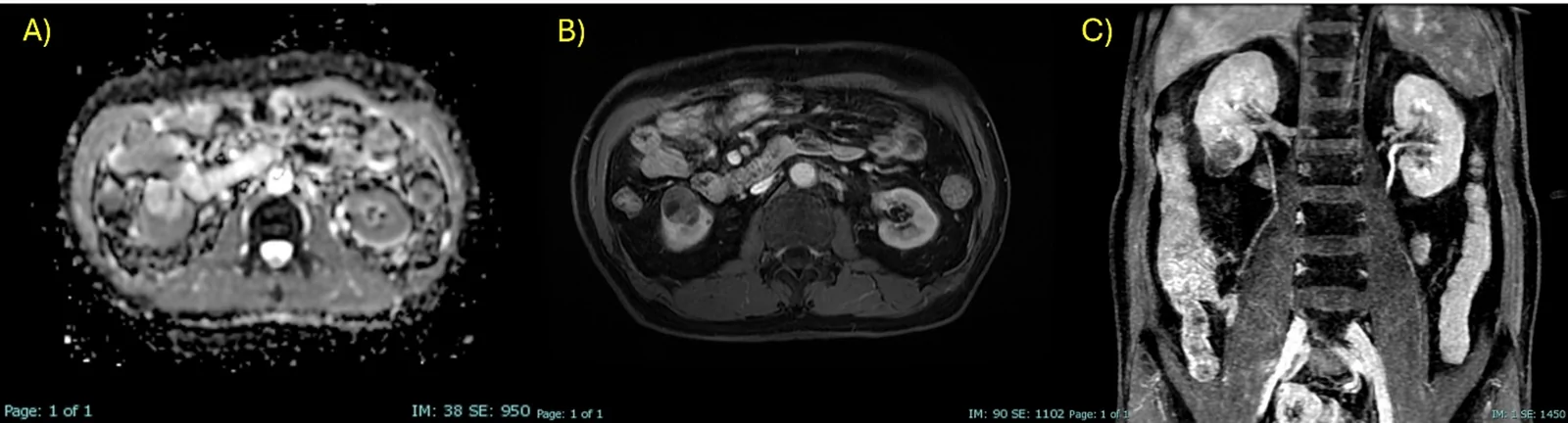
MRI images: axial apparent diffusion coefficient (ADC) map (**A**) and contrast-enhanced T1-weighted sequences acquired on axial (**B**) and coronal (**C**) planes showed indeterminate signal characteristics of the mural component

Gadolinium-enhanced MRI may be limited in CKD due to concerns about nephrogenic systemic fibrosis (NSF). Although macrocyclic agents have markedly reduced NSF risk and contemporary ESUR Contrast Media Safety Committee guidance supports cautious use of low-risk (macrocyclic/group II) agents in stage 4–5 CKD under selected circumstances, in many centers gadolinium administration at low eGFR remains selective rather than routine. When contrast-enhanced MRI is considered, the decision is typically patient- and context-specific (e.g., hemodynamic status, expected diagnostic gain, oncological urgency), using an appropriate macrocyclic agent and the lowest effective dose. When contrast is withheld, MRI's ability to assess enhancement, critical for differentiating benign from malignant lesions, is limited. DWI may offer supplementary information, but fibrosis can mimic restricted diffusion, reducing specificity [12, 15] (Figs. 3 and 4).

MRI thus retains an important role for specific indications, fat-poor angiomyolipoma assessment, venous invasion, or problem-solving, but its limitations in CKD necessitate alternative imaging options when vascularity must be definitively assessed.

**Table:** 

**What MRI can answer in CKD.** High soft-tissue contrast for lesion architecture, hemorrhage/protein content and fat-poor angiomyolipoma; DWI as an adjunct; evaluation of venous extension when indicated	
**Common MRI pitfalls in CKD.** Intrinsic T1 hyperintensity of hemorrhage/protein and heterogeneous CKD parenchyma can mimic solid components; fibrosis/inflammation may lower ADC and reduce DWI specificity; lack of gadolinium limits enhancement assessment	
**When to escalate to CEUS/biopsy.** Escalate to CEUS when MRI is non-contrast or enhancement remains equivocal; consider biopsy when CEUS shows an enhancing target but histology is needed for management, or when both MRI and CEUS remain indeterminate	

### Biopsy

Percutaneous renal mass biopsy provides valuable histological confirmation and can differentiate papillary RCC, oncocytoma, fat-poor angiomyolipoma and inflammatory pseudotumours. However, CKD kidneys are smaller, more fibrotic and often cystic, increasing technical difficulty and reducing diagnostic yield. Additionally, uremic platelet dysfunction increases bleeding risk. Biopsy is therefore reserved for cases where imaging remains indeterminate and the result would meaningfully influence management [16, 24, 31].

In patients with CKD, the decision to perform renal mass biopsy is particularly complex. Reduced kidney size, diffuse fibrosis and the high prevalence of cystic lesions increase sampling error, while uremia-related platelet dysfunction raises bleeding risk. In this context, CEUS may play a clinically relevant gatekeeping role by refining lesion selection for biopsy. By reliably differentiating truly vascularized mural nodules from avascular hemorrhagic or proteinaceous cystic components, CEUS can help identify lesions in which biopsy is most likely to be diagnostic and clinically impactful, while potentially reducing unnecessary or non-diagnostic procedures [24].

**Table:** 

**What it can answer in CKD.** Histological diagnosis and subtype to support risk stratification and tailor active surveillance, ablation or nephron-sparing surgery	
**Common pitfalls in CKD.** Lower diagnostic yield and sampling error in small, fibrotic or cystic kidneys; higher bleeding risk due to uremic platelet dysfunction; non-diagnostic results may still require follow-up imaging	
**When to escalate to CEUS/biopsy.** Use CEUS as a gatekeeper before biopsy to target truly enhancing mural/solid components and avoid avascular debris; proceed to biopsy when imaging (including CEUS) remains indeterminate and the result will meaningfully change management	

### Contrast-enhanced ultrasound

CEUS has become an increasingly important component of renal imaging, particularly for patients with CKD, in whom structural distortion and perfusional impairment hinder the performance of conventional modalities. CEUS employs gas-filled, purely intravascular microbubble contrast agents that are eliminated via the lungs and therefore do not pose nephrotoxic risk. This pharmacokinetic profile enables its safe application across all CKD stages, including dialysis-dependent patients, and circumvents the limitations associated with iodinated and gadolinium-based contrast agents [9, 20].

**Table:** 

**What it can answer in CKD.** Real-time microvascular perfusion to confirm or exclude enhancement, refine (CEUS-adapted) Bosniak categorization and distinguish enhancing mural nodules/septa from avascular hemorrhage or debris	
**Common pitfalls in CKD.** Operator dependence and limited acoustic windows (obesity, deep location or bowel gas); CEUS does not provide nodal or distant staging	
**When to escalate to CEUS/biopsy.** Escalate to biopsy when CEUS demonstrates an enhancing solid component and histology is required, or when CEUS is technically inconclusive; obtain CT/MRI (with an individualized contrast strategy) for staging once malignancy is suspected/confirmed	

The cumulative limitations of US, CT and MRI in CKD, especially their reduced ability to safely and reliably assess vascularity, provide the rationale for considering CEUS as an adjunctive problem-solving tool. Contemporary EFSUMB and WFUMB recommendations (2023–2024) endorse CEUS for the characterization of indeterminate renal masses, complex cystic lesions and focal renal abnormalities in CKD, as well as for surveillance scenarios where repeated contrast-enhanced CT or MRI would be inappropriate [14, 20].

Microbubble agents remain strictly intravascular, allowing real-time visualization of microvascular perfusion with excellent spatial and temporal resolution. This capacity to detect subtle enhancement is particularly valuable in hypoperfused CKD kidneys, where CT and MRI often produce equivocal results [32].

Reported diagnostic performance of CEUS for indeterminate renal masses is high, with sensitivity commonly reported between approximately 85–98% and specificity between 70 and 90% in published series, particularly for complex cystic lesions [23, 25, 32]. Several studies also describe Bosniak category reclassification rates of around 20–40% following CEUS evaluation, most frequently reflecting downgrading of lesions initially overclassified on CT due to hemorrhagic or proteinaceous cystic content [25, 32]. In CKD populations, CEUS may therefore provide a more reliable assessment of microvascular enhancement than non-contrast CT or MRI, where perfusion impairment and intrinsic signal alterations may limit diagnostic specificity [9, 23, 32].

**Fig. 5:**
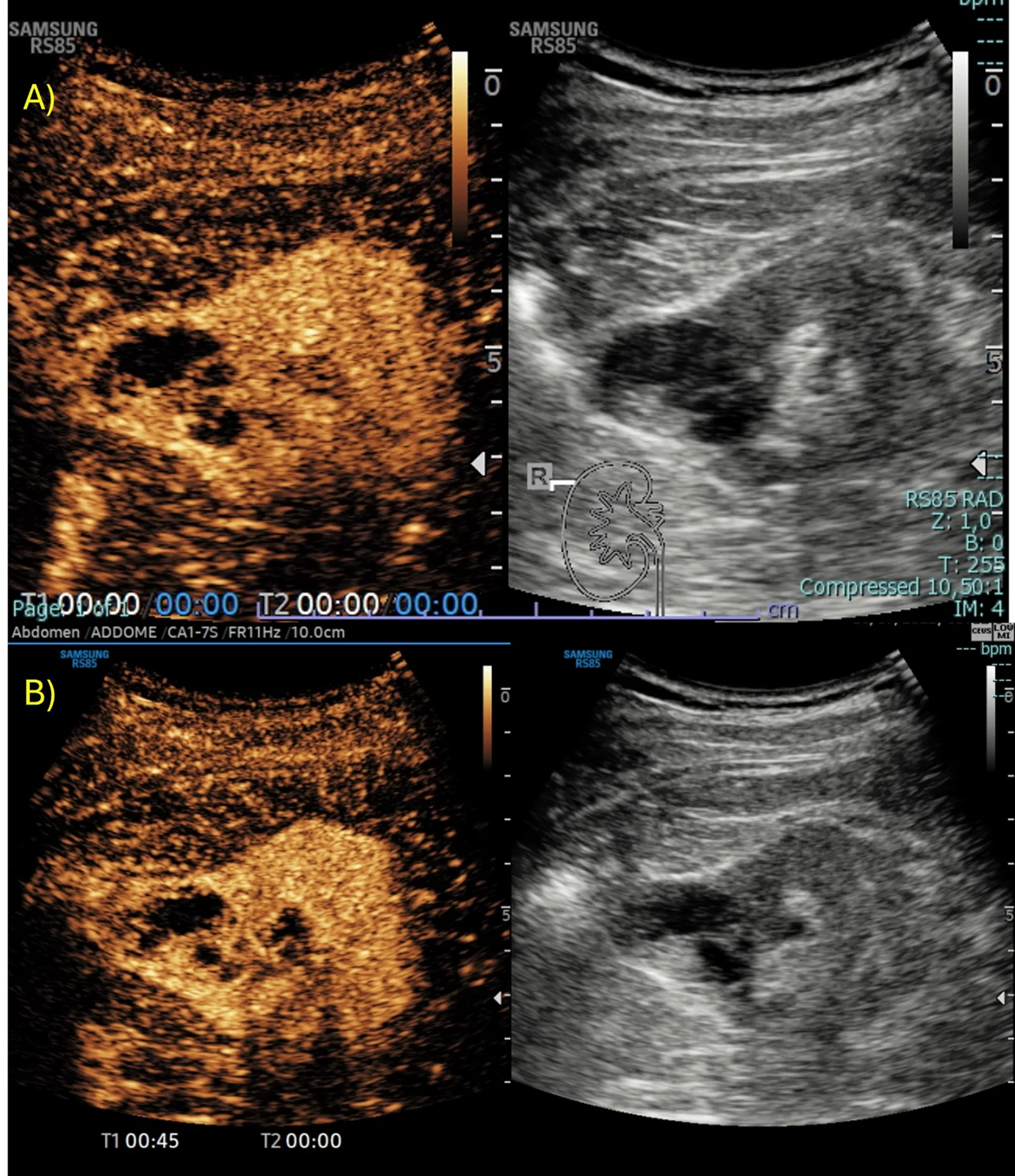
CEUS images acquired immediately after contrast administration (**A**) and at 45 s (**B**) showed focal contrast enhancement of the mural solid component, while the remaining cystic portion showed no enhancement

**Fig. 6:**
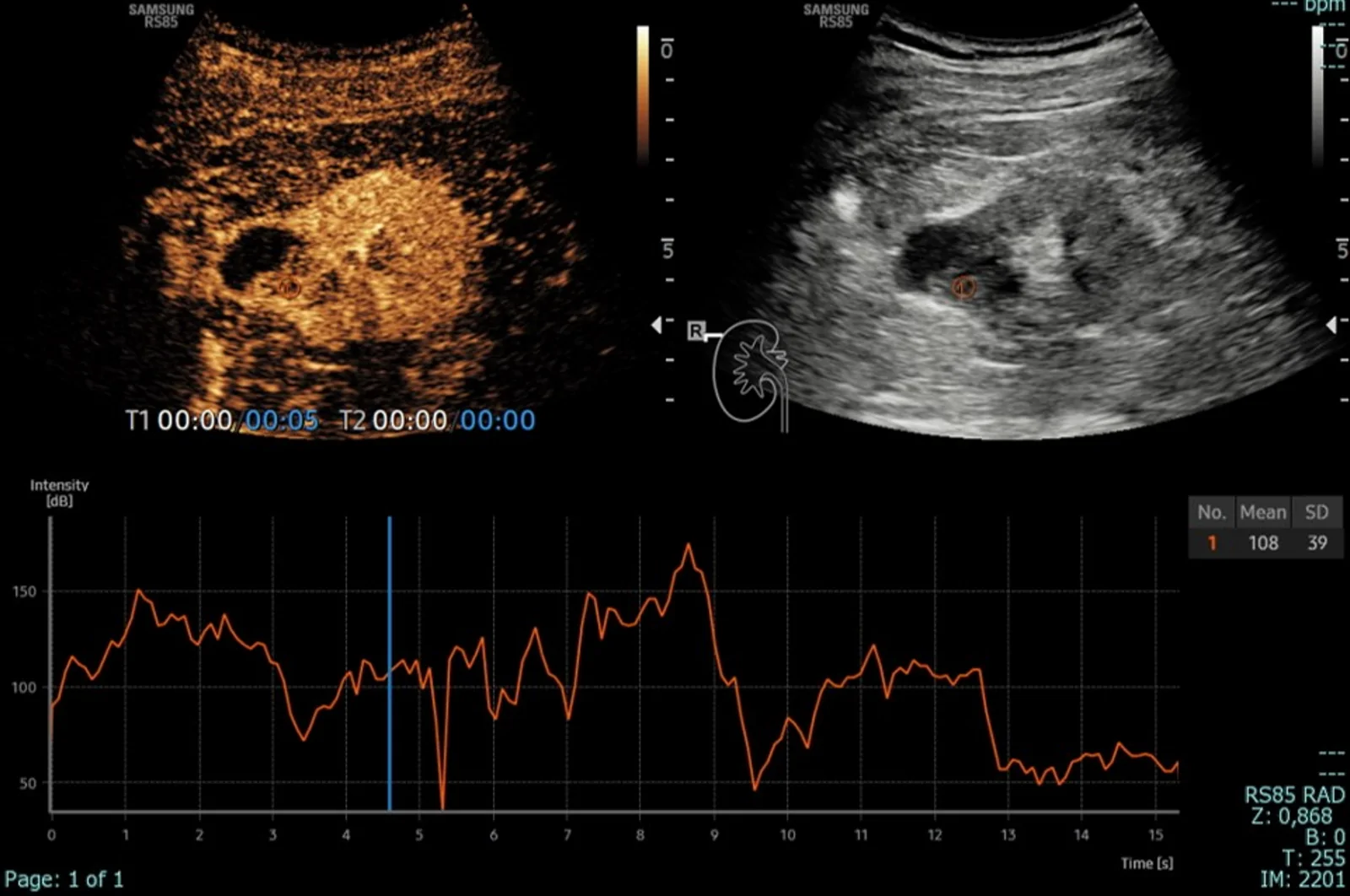
Quantitative CEUS analysis with time–intensity curve obtained from a region of interest placed over the mural component shows an early increase in contrast-specific signal intensity after contrast administration, followed by a peak and subsequent gradual signal decrease over time

CEUS provides real-time, high-resolution assessment of microvascular perfusion. This allows reliable distinction between enhancing tumor components and avascular hemorrhagic or proteinaceous material. It is particularly valuable in ACKD, where complex cysts may contain debris, thickened septa or hemorrhage that mimic solid elements on grey-scale US or non-contrast CT/MRI. Reduced cortical perfusion in CKD may also attenuate enhancement on CT, leading to overclassification within the Bosniak system. CEUS overcomes these limitations by demonstrating vascularized mural nodules clearly while leaving avascular structures unenhanced, often allowing reclassification of lesions initially labelled Bosniak IIF or III [23, 25] (Figs. 5 and 6).

CEUS also refines the assessment of solid renal masses. Malignant lesions often display rapid, heterogeneous wash-in followed by variable wash-out, whereas benign entities such as lipid-poor angiomyolipoma, oncocytoma or inflammatory pseudotumours tend to enhance more homogenously and persistently. The ability to observe these dynamic perfusion patterns in real-time provides diagnostic clarity in kidneys where CT and MRI may produce equivocal findings due to impaired perfusion [21, 22, 33].

Beyond initial characterization, CEUS is increasingly used for surveillance of small renal masses and for post-ablation follow-up, as its non-nephrotoxic profile permits repeated examinations without cumulative risk. EFSUMB guidelines emphasize CEUS as a preferred modality for monitoring indeterminate lesions and confirming ablation success [21, 22, 27].

CEUS is also valuable in renal transplant recipients, aiding in the detection of perfusion abnormalities, focal lesions and postoperative complications when conventional contrast-based imaging is unsuitable. Although transplant imaging lies outside the main focus of this review, its inclusion in recent guidelines underscores the broader relevance of CEUS in populations with impaired renal function [25, 34].

A summary of the main EFSUMB recommendations for renal CEUS applications is provided in Table 1.

**Table 1:** Summary of EFSUMB Recommendations for Renal CEUS

EFSUMB guideline	CEUS recommendation
Characterization of indeterminate renal lesions	CEUS recommended to assess vascularity, confirm enhancement, and differentiate solid masses from pseudo-lesions
Follow-up of renal lesions under active surveillance	CEUS recommended monitoring morphology and enhancement changes over time
Characterization of indeterminate lesions in transplanted kidneys	CEUS recommended evaluating focal graft lesions and differentiating inflammation, infarction, and tumor
Inflammatory–infectious pathologies	CEUS recommended to distinguish focal pyelonephritis from abscess and identify avascular collections
Advanced renal lesions and post-surgical bed assessment	CEUS indicated for assessment of venous extension (renal vein/inferior vena cava) and to distinguish recurrent tumor (enhancing) vs postoperative fibrosis (avascular)

Despite its strengths, CEUS is subject to certain limitations. It remains operator-dependent, with diagnostic accuracy reliant on optimal acquisition of cine loops, and acoustic windows may be suboptimal in obese patients or when kidneys are deeply located. Importantly, CEUS cannot stage lymph nodes or distant metastases, nor can it replace CT or MRI for comprehensive staging or assessment of extra-renal disease.

In summary, CEUS provides a robust, non-nephrotoxic and dynamically informative approach to renal mass evaluation in CKD. Its ability to sensitively depict microvascularity, overcome the interpretative limitations of CT and MRI and support reliable longitudinal surveillance makes it an essential modality within contemporary renal imaging pathways. Importantly, CEUS should be regarded as a complementary technique rather than a replacement for cross-sectional imaging, and its findings must be interpreted within a multimodality diagnostic framework.

## Discussion

### Imaging implications of an illustrative case in CKD

The presented case is used to illustrate the proposed CEUS-centered diagnostic workflow for indeterminate renal lesions in CKD and to demonstrate how each step of the algorithm addresses specific and recurrent sources of diagnostic uncertainty encountered with conventional imaging (Table 2).

**Table 2:**
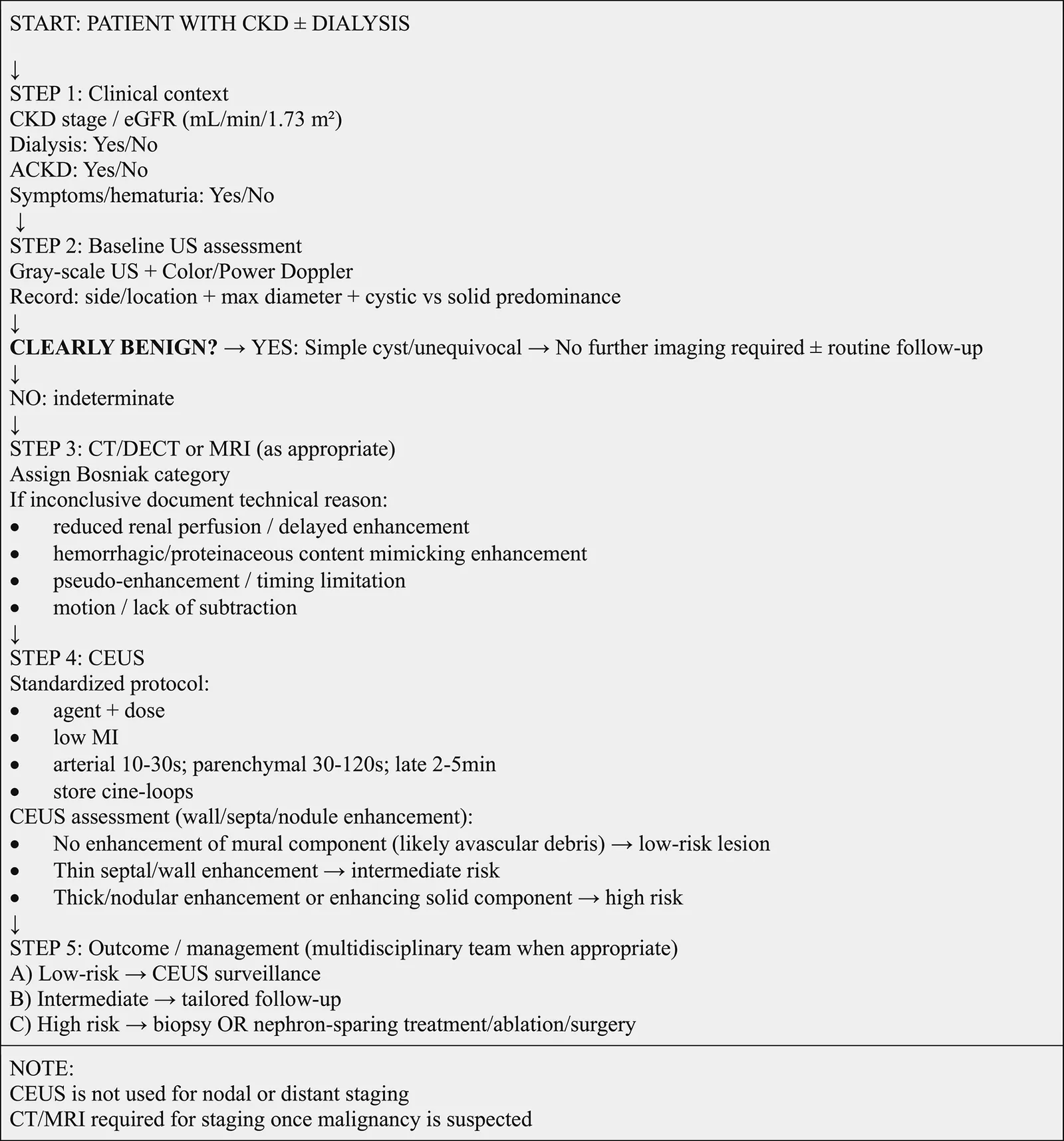
Mini-algorithm (CEUS-centered workflow in CKD)

Rather than representing an isolated scenario, this case reflects a common clinical situation in patients with advanced CKD and ACKD, in whom standard imaging pathways frequently fail to provide definitive lesion characterization.

At baseline assessment, grey-scale US and Color Doppler depicted a mural structure within a predominantly cystic renal lesion but did not demonstrate intralesional vascularity. This finding is typical in CKD kidneys, where reduced cortical perfusion and slow microcirculation limit Doppler sensitivity and may obscure true vascularized components. As outlined in the proposed workflow, such findings prompt escalation to cross-sectional imaging when lesions are not unequivocally benign.

DECT suggested faint iodine uptake within the mural component; however, interpretation remained inconclusive. Hyperattenuating hemorrhagic or proteinaceous cyst content, which is frequent in ACKD, can mimic enhancement on CT and DECT and artificially elevate Bosniak categorization. MRI contributed additional morphological and tissue characterization, but intrinsic T1 hyperintensity of cyst contents and equivocal DWI findings remained non-specific, particularly in the absence of contrast-enhanced sequences. Overall, persistent diagnostic ambiguity across CT/DECT and non-contrast MRI reflected the convergence of three well-recognized factors in CKD: attenuated or delayed enhancement due to hypoperfusion, cystic content mimicking enhancement, and technical limitations such as pseudo-enhancement or suboptimal timing.

According to the proposed workflow, once baseline imaging remains indeterminate and a dominant technical reason for inconclusiveness is identified, CEUS is positioned as the next problem-solving step to directly assess microvascular perfusion. In this case, CEUS enabled real-time evaluation of enhancement without nephrotoxic exposure.

CEUS was performed using a second-generation microbubble agent (e.g., sulfur hexafluoride or perflubutane), administered as an intravenous bolus (typical dose 1.0–2.4 mL, according to local practice) followed by a 5–10 mL saline flush. Low-mechanical-index imaging was used (MI typically ≤ 0.10) to minimize microbubble disruption. Cine-loops were continuously recorded from injection through the arterial phase (≈10–30 s), early parenchymal/corticomedullary phase (≈30–120 s) and late phase (≈2–5 min), with key still frames stored. Enhancement assessment was used to evaluate wall or septal thickening, mural nodules and solid components and, in this case, demonstrated enhancement of the mural component with no enhancement of the cystic portion.

These findings resolved the residual ambiguity of prior imaging by confirming the presence of a vascularized mural component while excluding enhancement of the cystic contents, a distinction that could not be made reliably with CT/DECT or non-contrast MRI in this hypoperfused CKD kidney. This clarification directly informed downstream management while avoiding exposure to iodinated or gadolinium-based contrast agents.

### Clinical impact and health-system implications

In patients with CKD, the diagnostic evaluation of renal masses has direct implications not only for lesion characterization, but also for downstream clinical management and resource utilization. In this setting, CEUS may offer a practical advantage by improving the assessment of indeterminate renal lesions without exposing patients to nephrotoxic iodinated contrast agents or gadolinium-based contrast media. This may help reduce reliance on contrast-enhanced CT, particularly in patients with advanced renal impairment or in those requiring repeated imaging follow-up.

Improved lesion characterization with CEUS may also contribute to a more appropriate selection of cases for biopsy, surveillance, or intervention, potentially reducing unnecessary invasive procedures in selected patients. In addition, a more confident distinction between benign, indolent, and suspicious lesions may support multidisciplinary decision-making and facilitate nephron-sparing strategies whenever oncologically feasible, which is particularly relevant in patients with limited renal reserve.

From a health-system perspective, the use of CEUS in appropriately selected CKD patients may contribute to a more tailored and resource-conscious diagnostic pathway. However, these potential benefits should be interpreted with caution, as robust outcome-based evidence remains limited and prospective studies are needed to determine whether CEUS-based pathways effectively reduce biopsy rates, contrast-enhanced CT utilization, and treatment-related morbidity.

## Conclusions

Renal mass evaluation in CKD remains a major diagnostic challenge due to profound structural remodeling, perfusion deficits and limitations related to contrast agent use. These factors substantially reduce the reliability of conventional imaging, contributing to the high prevalence of indeterminate lesions in this population. In this context, CEUS offers a particularly suitable adjunctive solution. Its non-nephrotoxic, purely intravascular microbubble agents allow safe application at any CKD stage, overcoming the restrictions inherent to iodinated and gadolinium-based contrast media. By enabling real-time assessment of microvascular perfusion, CEUS provides diagnostic clarity in complex cystic lesions, hypovascular tumors and lesions obscured by the morphological distortions characteristic of CKD and acquired cystic kidney disease.

Contemporary EFSUMB, ESUR and WFUMB recommendations increasingly position CEUS as an essential component of renal mass imaging, both for initial characterization and for longitudinal surveillance. Its integration into clinical practice can reduce unnecessary follow-up imaging, minimize invasive procedures and support nephron-sparing strategies, which are particularly important in patients with limited renal reserve. As CKD prevalence rises globally, CEUS will play an expanding role in achieving safe, accurate and patient-centered renal tumor evaluation.

In clinical practice, a CEUS-centered diagnostic approach may help refine the evaluation of renal masses in CKD, reduce the need for contrast-enhanced CT in selected patients, and support management decisions including surveillance, biopsy, or nephron-sparing treatment. Nevertheless, this proposed workflow remains conceptual and should be validated in prospective studies assessing its impact on diagnostic accuracy, patient outcomes, and health-care resource utilization.
